# Harnessing Microalgae and Cyanobacteria for Sustainable Pesticide Biodegradation: Advances, Challenges, and Ecological Benefits

**DOI:** 10.3390/microorganisms13102404

**Published:** 2025-10-21

**Authors:** Nurziya R. Akmukhanova, Sandugash N. Seiilbek, Bolatkhan K. Zayadan, Kenzhegul Bolatkhan, Ramina A. Bakytzhan, Gulzhaina S. Domash, Barry D. Bruce

**Affiliations:** 1 Department of Biotechnology, Faculty of Biology and Biotechnology, Al-Farabi Kazakh National University, Almaty 050040, Kazakhstan; sseilbek1@gmail.com (S.N.S.); zbolatkhan@gmail.com (B.K.Z.); bkenzhegul23@gmail.com (K.B.); ramina.baytzhan@mail.ru (R.A.B.); domashg28@mail.ru (G.S.D.); 2 Department of Biochemistry, Cellular and Molecular Biology, University of Tennessee, Knoxville, TN 37996, USA; 3 Department of Microbiology, University of Tennessee, Knoxville, TN 37996, USA

**Keywords:** microalgae, cyanobacteria, biodegradation, pesticide, soil health

## Abstract

Microalgae and cyanobacteria, as versatile photoautotrophic microorganisms, hold significant promise for mitigating soil and water pollution—particularly the removal of pesticides. This review examines their multifaceted roles in pesticide biodegradation, emphasizing how their metabolic capabilities simultaneously reduce environmental toxicity, enrich soil properties, and support beneficial microbiota. Cultivation in wastewater treatment systems further highlights their potential for cost-effective bioremediation, as these microbes degrade pesticides, recycle nutrients, break down organic pollutants, and generate biomass with value-added applications. Despite these advantages, implementing large-scale processes remains challenging. Key hurdles include optimizing growth parameters, preventing contamination, improving harvest efficiency, and designing robust bioreactors. Addressing these complexities demands interdisciplinary collaboration in strain selection, metabolic engineering, and process intensification. By capitalizing on microalgae and cyanobacteria’s adaptability and metabolic flexibility, we can develop more sustainable management strategies that reduce reliance on chemical inputs, foster soil health, and contribute to long-term ecological restoration. Ultimately, these microorganisms have the potential to reshape environmental stewardship by combining economic viability with broad-scale ecological benefits.

## 1. Introduction

Intensive pesticide use has undoubtedly bolstered agricultural productivity and minimized crop losses, thus contributing to global food security and economic growth. However, their persistent nature and high accumulation rates pose severe environmental risks with frequent application [1]. According to forecasts by Statista, global agricultural pesticide consumption is expected to rise to 4.41 million metric tons by 2027 [2]. Despite growing interest in organic methods, chemical pesticides remain integral for enhancing crop yields and ensuring regional food security. Yet, awareness of these chemicals’ health and ecological dangers has led to increased efforts to mitigate their negative impacts [3]. Adhering to international standards for managing highly hazardous pesticides remains challenging. During the “Green Revolution,” synthetic pesticides were widely adopted to boost productivity and meet global food demands; however, this approach harmed ecosystems and human health. These unfavorable outcomes ultimately drove a shift toward sustainable development in the 1960s, prioritizing economic growth, societal acceptance, and environmental protection.

In response, a comprehensive framework of 17 Sustainable Development Goals (SDGs) and 169 related targets was established [4]. Some SDGs focus on eradicating hunger, safeguarding human health, restoring ecosystems, and combating climate change. Achieving these objectives necessitates the responsible use of chemicals and the preservation of soil and water quality. Bioremediation stands out as a promising strategy to rehabilitate pesticide-contaminated soils by leveraging the metabolic capabilities of microorganisms, particularly microalgae [5]. However, compared to bacteria and fungi, microalgae remain relatively underexplored in soil bioremediation, despite growing interest in their potential applications. Harnessing these biological resources within the soil biocenosis can expedite the remediation and recovery of affected ecosystems. As ecological engineers, microalgae and cyanobacteria regulate soil processes and create habitats for other organisms [6].

Their multifaceted contributions to soil health include forming organic matter through biochemical reactions and colonizing niches unoccupied by higher plants, thereby capturing additional solar energy and producing supplementary biomass, which improves soil structure, enhances nutrient availability, and supports microbial diversity, ultimately promoting soil fertility and ecosystem resilience [7]. These organisms are critical in early-stage soil development and influence soil’s physical and chemical properties. Their functional repertoire includes improving soil fertility through organic matter production, nitrogen fixation, and enhanced nutrient availability, as well as providing anti-erosion benefits via mucilaginous substances and filamentous thalli [8,9,10]. By incorporating such sustainable approaches to pest management and soil health, agriculture can move closer to achieving key global sustainability objectives while mitigating the far-reaching impacts of pesticide misuse.

The use of microalgae and cyanobacteria in bioremediation is advantageous because they can continue to grow even after the primary contaminant (organic pollutant) source is depleted, thanks to mixotrophic metabolism—combining photosynthesis with uptake of organic carbon. This contrasts with strictly heterotrophic bacteria, whose growth often declines when contaminant concentrations drop [11]. However, this capacity may be limited in deeper soil layers due to reduced light penetration, which restricts photosynthetic activity. This highlights the urgent need to identify highly adaptable strains of these photoautotrophic microorganisms that can efficiently degrade organic pollutants while simultaneously improving soil fertility. Although microalgae are frequently present in contaminated habitats, their role in soil pollutant degradation is often underestimated. Consequently, investigating microalgae and cyanobacteria with pesticide biodegradation capabilities is critical not only for remediating contaminated ecosystems but also for reducing the environmental impacts of pesticide use in agriculture.

This article aims to evaluate the potential of microalgae and cyanobacteria in pesticide biodegradation and soil fertility enhancement while examining their incorporation into sustainable farming practices. Specifically, it explores the mechanisms of pesticide breakdown, the impact of these microorganisms on soil structure and nutrient availability, and their overall economic and environmental benefits. The novelty of this work lies in its interdisciplinary perspective, integrating information on the metabolic pathways of pesticide degradation, the ecological ramifications of using microalgae and cyanobacteria, and their contributions to biomass production in closed-loop agroecosystems. Synthesizing these findings, the study advances environmentally sound strategies for managing agricultural landscapes and mitigating agrochemical pollution.

To achieve these objectives, the review is based on a systematic selection of literature from international databases including Scopus, Web of Science, PubMed, and Google Scholar. The search was conducted using the keywords “Microalgae,” “Cyanobacteria,” “Biodegradation,” “Pesticide,” and “Soil Health.” Preference was given to peer-reviewed publications from the last 10–15 years that directly address the role of microalgae and cyanobacteria in pesticide degradation and soil fertility improvement.

## 2. The Problem of Soil Contamination with Pesticides

The global expansion of agriculture has driven a sharp rise in pesticide use, conferring considerable economic gains yet posing serious threats to environmental health and human well-being. Pesticides are purposefully introduced to control pests and safeguard crops, thereby boosting yields and enhancing the quality of agricultural commodities [12]. They offer direct benefits—reducing pest-related damage—and indirect advantages such as increased income and improved living standards [13]. Beyond farming, pesticides help maintain recreational areas and protect public health, anchoring their role in the global economy. However, the intensifying reliance on chemical controls underscores the urgent need to reconcile agricultural productivity with environmental stewardship.

Modern irrigation techniques, optimized seed varieties, and the judicious application of agricultural chemicals have collectively quadrupled grain yields since the late 17th century [14]. Effective weed management, mainly through pesticide use, has been instrumental in preventing substantial crop losses. For example, in medium-level rice cultivation, timely and efficient weed control can avert yield reductions of 28% to 48%, while severe infestations can result in up to a 40% decrease [15]. Although these achievements have bolstered global food security, the intensification of pesticide application raises significant concerns about sustainability and the long-term health of ecosystems.

**Figure 1:**
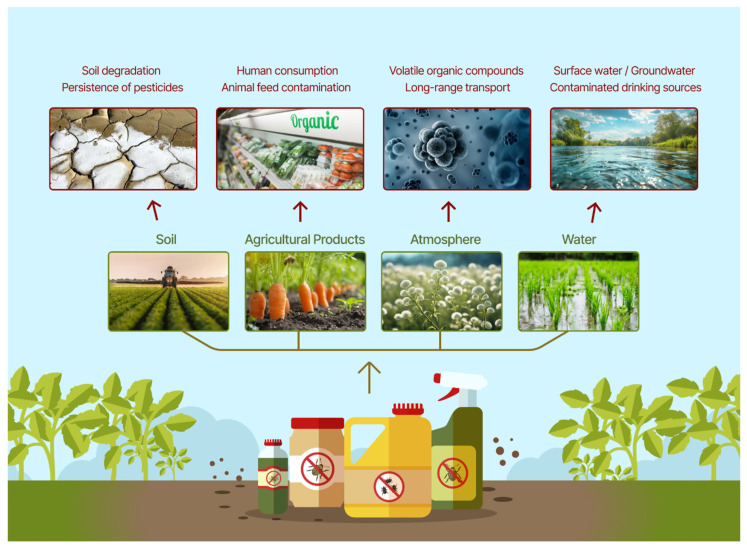
Illustration of possible ways of pesticide exposure to the environment.

The pervasive use of pesticides carries critical risks, as these compounds contaminate soil, water, air, and food supplies, jeopardizing both biodiversity and public health. Once released, their distribution and transformation escape direct human oversight, compounding challenges in monitoring and mitigation (Figure 1). Different classes of pesticides exert specific toxic effects: organophosphates disrupt acetylcholinesterase activity, leading to neurotoxicity [16]; organochlorines persist in the environment and bioaccumulate, causing endocrine disruption and reproductive toxicity [17]; carbamates also inhibit cholinesterase but are less persistent, producing acute but reversible neurotoxic effects [18]; and triazine herbicides interfere with photosynthesis in plants and can act as endocrine disruptors in animals [19]. Environmental factors—such as soil chemistry, oxygen availability, light intensity, and climate—further influence how pesticides degrade, disperse, and accumulate in living organisms. Alarmingly, many pesticides form novel byproducts with unique chemical and biological properties, which can be more toxic than the primary compound. For example, the herbicide atrazine degrades into deethylatrazine and deisopropylatrazine, both of which are more mobile in soil and groundwater and may disrupt endocrine systems [20]. Similarly, the abiotic transformation of the insecticide chlorpyrifos in chlorinated water can lead to the formation of chlorpyrifos oxon, a metabolite with significantly greater acetylcholinesterase inhibitory activity and, consequently, higher toxicity than the primary compound [21]. Given the far-reaching ecological and socioeconomic ramifications, addressing the buildup of pesticides in the soil is an increasingly urgent priority for global agriculture.

Pesticides profoundly negatively impact wildlife, particularly birds and other non-target organisms. In the United States alone, an estimated 72 million birds die yearly from pesticide exposure [22]. These chemicals can trigger acute and chronic effects, influencing wildlife at cellular, organismal, and population levels [23]. As growing populations and agricultural demands drive increased pesticide use, global biodiversity faces escalating threats. Although the mechanisms by which pesticides target pest species are often well characterized, their unintended consequences for wildlife and ecosystems remain insufficiently examined [24].

Despite the notable economic advantages of pesticides, their adverse effects on human health and the environment are a source of heightened concern. Pesticides can enter the human body through inhalation, ingesting contaminated food or water, and skin contact [25]. The skin represents a major route of pesticide entry, particularly during production and application or through environmental contamination [26,27]. Residual pesticides have been detected in water, air, soil, and various living organisms, underscoring their pervasive presence [25].

Health effects stemming from pesticide exposure include both acute symptoms (e.g., abdominal pain, dizziness, headaches) and chronic conditions such as cancer, leukemia, and asthma [25]. The degree of risk is influenced by factors like toxicity level, exposure duration, and individual susceptibility, with children, pregnant women, and the elderly being particularly vulnerable [25]. Although pesticides confer benefits in agriculture and public health, their widespread use continues to spark debates regarding potential harm to human health and ecosystems [27].

In addition to direct health risks, pesticides pose significant environmental hazards by contaminating air, soil, and water resources [28]. They enter the atmosphere through aerial spraying and volatilization, sometimes accumulating overtime. Pesticide residues can disturb microbial communities, alter chemical properties, and harm beneficial invertebrates, reducing soil fertility [29]. Aquatic systems are especially susceptible to contamination through direct spraying and runoff, which can pollute streams, lakes, and inland waters [28]. Of particular concern are persistent compounds like organochlorines, which accumulate in food chains and amplify environmental and health risks [30].

The pervasive use of pesticides poses serious challenges, including groundwater and food contamination and the bioaccumulation of toxic substances in plants and animals. To mitigate these risks, the Pesticide Action Network (PAN) has compiled a comprehensive list of highly hazardous pesticides [31] to prevent pesticide misuse and promote environmentally friendly alternatives. One promising strategy in this regard is the expansion of organic farming, which provides sustainable pest control measures and enhances the overall environmental performance of agriculture. Economic considerations largely explain the industry’s preference for conventional farming, as higher wages and stable yields make it more attractive despite known health risks [32]. Moreover, economic analyses indicate that pesticide use in conventional systems is close to optimal, reinforcing their predominance over organic practices that rely primarily on machinery and cultural changes [33]. Nevertheless, recent meta-analyses and long-term field trials show that although organic farming yields remain on average ~18–25% lower than conventional systems, this gap can be significantly narrowed by improved agronomic practices and region-specific management [34,35]. In some cases, organic farms have achieved comparable economic performance to conventional ones, especially when environmental and social co-benefits are included [36].

Various laws and international agreements regulate pesticide use to minimize their impact on human health, crops, and ecosystems. Three legally binding global conventions—the Stockholm Convention [37], the Rotterdam Convention [38], and the Montreal Protocol [39]—specifically address persistent organic pollutants (POPs). Their goal is to limit the harmful effects of these chemicals on humans and the environment. Methyl bromide, for instance, is governed by the Montreal Protocol due to its ozone-depleting properties. In 2009, a joint FAO/WHO expert group confirmed that pesticides covered under these conventions belong to the POPs category, subjecting them to special international attention and regulatory measures [40].

Pesticide regulation and approval differ widely across major agricultural nations [41]. Globally, there is a pressing need for improved pesticide governance to balance food production demands with environmental stewardship [42]. This includes more robust monitoring of pesticide impacts at the landscape level and proactive efforts to ban harmful substances, incorporating new approaches resembling those used in pharmaceutical oversight [42].

Reducing pesticides’ adverse effects on human health and ecosystems requires a comprehensive strategy. Such an approach involves the safe storage and disposal of pesticide waste and the remediation of contaminated sites. Advances in soil restoration technologies are vital for ensuring environmental safety, demanding tailored solutions based on specific site characteristics and pollutant profiles. Effective remediation extends beyond purifying the soil to prevent contaminants’ spread, underscoring the importance of regular soil and water quality monitoring and establishing buffer zones around agricultural areas.

Interdisciplinary collaboration is essential for remediating polluted sites and integrating biological and chemical methods. Alongside the chemical breakdown of pesticides, restoring soil microbiota is pivotal for maintaining healthy ecosystem function. Biodegradation has emerged as an innovative and promising strategy for detoxifying pesticide-contaminated areas [43]. This process capitalizes on microorganisms capable of degrading toxic compounds into less harmful or inert substances, thereby reducing environmental and health risks [44]. Current research aims to identify and optimize microbial strains effective against a range of pesticides and to develop scalable technologies that can restore extensive contaminated regions [45]. Beyond its ecological benefits, biodegradation also decreases the need for chemical decontamination methods, potentially offering more sustainable and cost-effective solutions for managing pesticide pollution.

## 3. Microalgae as Pesticide Biodegraders: Mechanisms and Degradation Processes

Microalgae comprise a highly diverse group of microscopic photosynthetic organisms, encompassing prokaryotic cyanobacteria and eukaryotic green and diatom algae [46]. Their capacity to transform CO_2_ into biomass and various bioproducts—ranging from nutrients to biofuels—has fueled applications in bioremediation, biofuel production, and even the cosmetics and pharmaceutical industries [47,48]. Within this broad scope, one particularly critical use lies in pesticide removal from soil and water. As ubiquitous photosynthetic microorganisms found in both aquatic and terrestrial habitats, microalgae are capable of utilizing autotrophic, mixotrophic, and heterotrophic modes of metabolism and excel at breaking down organic and inorganic pollutants [8]. They have also shown promise in remediating industrial wastewater contaminated with pesticides, removing toxic compounds, metals, excess nutrients, and atmospheric CO_2_ [7,49,50] (Table 1).

Two primary processes are central to these remediation capabilities: biosorption and biodegradation [58]. Biosorption is a rapid, energy-independent mechanism involving the passive binding of pesticides onto microalgal cell walls—a process facilitated by functional groups like carboxyl, hydroxyl, and amine moieties [49,59,60]. Modifications in cell wall structure, pH, temperature, and competing ions can significantly affect this adsorption efficiency [61,62,63]. Biosorption using microalgae, particularly *Chlorella* species, demonstrates significant potential for pesticide removal from contaminated water. Studies have shown that *Chlorella vulgaris* can remove 86–99% of pesticides, with dead or lyophilized biomass often outperforming living cells [59,64]. Similarly, *Chlorella sorokiniana* achieved up to 99.8% removal of chlorpyrifos at 300 ppm within 120 h [65]. These results indicate that biosorption, primarily through adsorption onto algal cells, is highly effective, although efficiency depends on factors such as pH, contact time, and initial concentrations [66]. For example, an increase in pH leads to decreased biosorption efficiency, as many pesticides under these conditions may exist in anionic form that binds less effectively to cell walls [7]. Moreover, coexisting contaminants such as heavy metals (Pb^2+^, Cd^2+^, Zn^2+^) and organic pollutants may compete for the same binding sites (e.g., carboxyl, hydroxyl, and amino groups) on algal cell surfaces, thereby reducing biosorption efficiency and complicating real-world remediation processes [67].

Biodegradation complements biosorption by actively breaking down pesticides into less harmful or non-toxic substances. Biodegradation complements biosorption by actively breaking down pesticides into less harmful or non-toxic substances. This process leverages the metabolic versatility of microalgae, supported by antioxidant enzymes and other protective mechanisms that mitigate oxidative stress [49]. Collectively, these strategies enable microalgae to treat a variety of pollutants—including phosphorus, nitrogen, heavy metals, dyes, and pharmaceutical compounds—while thriving in diverse environments [50]. Combining biosorption’s rapid, passive uptake with biodegradation’s sustained metabolic breakdown, microalgae offer a multifaceted solution for pesticide remediation in agricultural runoff and industrial effluents, contributing to more sustainable and ecologically responsible pest management practices [5,49].

These complementary mechanisms highlight the significant potential of microalgae in reducing pesticide loads from contaminated environments [58]. Biosorption, in which pesticides are adsorbed onto cell walls or secreted substances, is a rapid process that occurs within minutes [59]. This approach involves the passive binding of pesticides to the cell walls of microalgae, making it a fast and energy-independent purification method. The adsorption of pesticides facilitates biosorption onto the surface of microalgal cells, where interactions with the cell walls are promoted by the presence of functional groups such as carboxyl, hydroxyl, and amine [49,60]. These groups serve as binding sites for pollutants, enabling microalgae to remove pesticides and heavy metals through biosorption [60]. The complex structure of microalgal cell walls and antioxidant enzymes play a crucial role in pesticide removal and protection against reactive oxygen species [49]. Spectroscopic analyses and chemical modification studies have identified carboxyl and amine groups as key functional moieties responsible for binding [61]. However, exposure to pollutants such as pesticides (herbicides and insecticides), hydrocarbons, and heavy metals can alter the cell wall structure, affecting pore size, surface area, and the composition of functional groups [62]. The chemical makeup of the cell wall strongly influences biosorption capacity—cell walls rich in polysaccharides, lipids, and proteins can adsorb significant amounts of pesticides [68].

One of the main advantages of biosorption is its speed and ease of implementation: in long-term experiments, *Chlorella vulgaris* demonstrated the removal of 87–96.5% of pesticides such as chlorpyrifos, malathion, and carbofuran, and up to 99% in short-term studies [59]. In agricultural runoff, *Chlorella* sp. achieved 100% removal of chlorpyrifos, while *Scenedesmus* sp. removed 75% [69]. However, despite the high efficiency of biosorption, this process is reversible when environmental conditions such as pH or temperature change, adsorbed pesticides may be released back into the water, necessitating further refinement of removal processes [59]. Moreover, other contaminants in the water can reduce effectiveness by competing with pesticides for binding sites on the surface of microalgal cells [67]. An important consideration is that pollutants adsorbed by microalgae may become available again if no further biodegradation occurs. Therefore, integration with bacteria and fungi capable of degrading pesticides is essential to ensure their complete detoxification [70]. While contaminated biomass can be harvested from aquatic environments, its application in soils requires careful management, since residual pollutants may be re-released [71]. In situ applications that combine biosorption with microbial biodegradation are particularly promising, as they allow for effective remediation while saving water and time at contaminated sites [72].

Biodegradation, which converts pesticides into simpler and less toxic compounds, is considered the most promising mechanism for long-term reclamation [58]. The efficiency of pesticide removal and its impact on microalgal metabolism depend on factors such as pesticide type, concentration, and the strain of algae used [49]. Through their metabolic processes, microalgae can break down organic substances into smaller molecules; some of these products can serve as nutrients to support their growth, while others may not be metabolized efficiently [73]. Notably, the efficiency of pesticide biodegradation by microalgae often surpasses that of photodegradation under natural lighting [74]. For example, three filamentous algal strains—*Nostoc muscorum*, *Anabaena oryzae*, and *Spirulina platensis*—showed average removal efficiencies for the organophosphorus pesticide malathion of 91%, 65%, and 54%, respectively [75]. In another study, five green algae species, including *Scenedesmus* sp. *MM1*, *Scenedesmus* sp. *MM2*, *Chlamydomonas* sp., *Stichococcus* sp., and *Chlorella* sp., demonstrated high detoxification abilities for fenamiphos, with *Chlorella* sp. achieving a removal efficiency of over 99% [76]. Microalgae can degrade pesticides through natural metabolic pathways, including enzymatic processes such as esterase, transferase, and cytochrome P450, converting them into less toxic organic compounds while releasing oxygen [77]. Various pesticides—including atrazine, propanil, metolachlor, pyriproxyfen, dimethoate, carbofuran, isoproturon, molinate, and simazine—can be degraded by microalgae up to 87–96% under controlled conditions [64]. For instance, at a trichlorfon concentration of 100 mg/L, *Chlamydomonas reinhardtii* showed 100% pesticide removal, although higher concentrations of 200 mg/L suppressed algal growth by 51% [78]. Two microalgal strains, *Microcystis* sp. and *Scenedesmus quadricauda*, demonstrated the ability to remove 18.3% and 15.2% of mesotrione, respectively, after 96 h of cultivation in BG-11 medium supplemented with mesotrione at 5 mg/L [79].

Microalgae possess catabolic genes that enable the breakdown of numerous soil pollutants [11]. They demonstrate considerable potential for bioremediation through multiple mechanisms. Microalgae efficiently remove heavy metals and pesticides from contaminated soils, providing advantages such as high remediation efficiency, low operational costs, and eco-friendly treatment compared to conventional chemical or physical approaches [80]. Indigenous microbial consortia in diesel-contaminated soils carry catabolic genes (e.g., *xylE*, *ndoB*) involved in aromatic hydrocarbon degradation, with a significant fraction of colonies harboring these degradative genes [51]. Mobile genetic elements carrying catabolic genes may accelerate bioremediation via horizontal gene transfer, although field-scale applications remain limited [81]. Mixotrophic cyanobacteria and microalgae possess endogenous enzymes such as cytochromes P450, glutathione-S-transferases, and esterases, which participate in the transformation of organic pollutants, combining carbon sequestration with degradative functions and making them promising candidates for genetic engineering and large-scale bioremediation applications [11]. Many species have been studied for their potential in bioremediation, with cyanobacteria such as *Anabaena* sp. *PCC7120* and *Nostoc ellipsosporum* show particular efficacy in degrading lindane, a persistent chlorinated pesticide [82]. The process involves dechlorination, converting lindane into simpler compounds such as pentachlorocyclohexene and trichlorobenzene [83]. This transformation is dependent on nitrate availability and the presence of a functional *nir* operon, which encodes the enzymes required for nitrate utilization [84]. *Anabaena* sp. *PCC7120* also exhibits the capacity to biotransform other pesticides (e.g., aldrin and chlorpyrifos-methyl) via cytochrome P450-dependent monooxygenases [85]. Recent studies point to the presence of potential *lin* gene orthologs in *Anabaena* sp. *PCC7120*, suggesting additional genetic pathways involved in lindane degradation [83].

Other microalgae and cyanobacteria likewise demonstrate robust pesticide-degrading abilities: *Chlorella vulgaris*, *Scenedesmus bijugatus*, *Nostoc linckia*, *Nostoc muscorum*, *Oscillatoria animalis*, and *Phormidium foveolarum* all actively metabolize the organophosphorus insecticide methyl parathion by using it as a phosphorus source [52]. *Anabaena fertilissima* has proven especially effective at exploiting pesticides for phosphorus [86]. *Anabaena* sp. can degrade lindane, converting it into γ-pentachlorocyclohexene [82], while soil cyanobacteria like *Chlorococcum* sp., *Anabaena* sp., and *Nostoc* sp. catabolize DDT to its metabolites DDD and DDE [87]. *Chlamydomonas reinhardtii* exhibits high tolerance toward trichlorfon, achieving complete biodegradation at concentrations up to 100 mg/L [78]. A mixed culture of *Chlorella vulgaris*, *Scenedesmus quadricauda*, and *Spirulina platensis* removes up to 99% of malathion and simultaneously reduces nickel, lead, and cadmium by 95%, 89%, and 88%, respectively [88]. Additionally, *Scenedesmus obliquus* can degrade chlorinated phenols at varying efficiencies tied to toxicity level [89]. Microalgae such as *Chlorella vulgaris* also convert diazinon into less toxic metabolites [90], while *Scenedesmus* sp. and *Chlorococcum* sp. transform endosulfan into endosulfan sulfate [91]. *Chlorella* sp. demonstrates high atrazine-degrading efficiency, particularly at lower concentrations (5 mg/L). This involves photocatalytic degradation, a process in which light energy drives enzymatic or reactive species-mediated breakdown of atrazine, achieving a photo-catabolic degradation coefficient of 31.4% within 60 min and removing 83.0–64.3% of atrazine at 40–80 μg/L after eight days [92].

**Figure 2:**
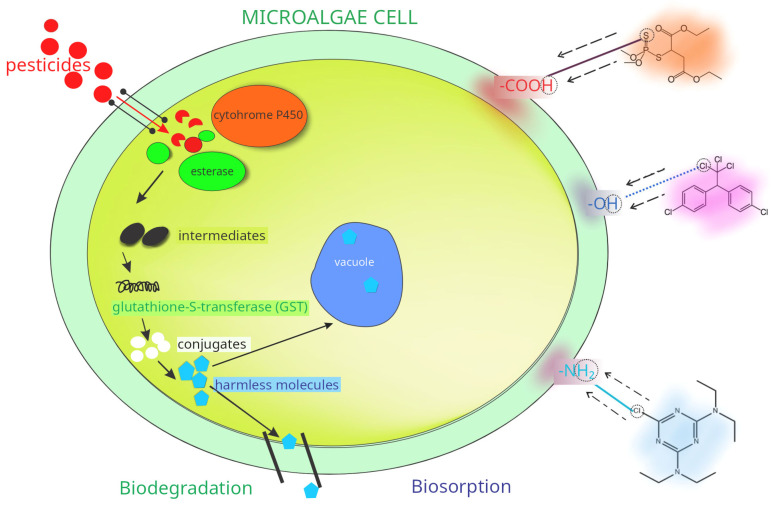
Natural Mechanisms of Pesticide Detoxification by Microalgae.

Beyond organism-level adaptations, metabolic pathways are crucial for pesticide biodegradation, as microorganisms rely on diverse enzymatic systems to break down these compounds into less toxic byproducts. Esterases, for instance, play a pivotal role in the initial hydrolysis of organophosphates, carbamates, and pyrethroids [93]. Carboxylesterases—a specialized class of these enzymes—are notably practical at detoxifying ester-based agrochemicals and occur in plants, animals, and microbes [94,95]. In addition, antioxidant enzymes such as superoxide dismutase, catalase, and ascorbate peroxidase facilitate pesticide elimination and shield cells from reactive oxygen species [49]. Carboxylesterase activity also underpins the degradation of organophosphates [96]. Although high pesticide levels can disrupt microalgal metabolic functions, strains like *Chlorella sorokiniana* can withstand concentrations of up to 100 ppm [96]. Cytochrome P450 enzymes further contribute to this process by mediating the oxidative degradation of xenobiotics, including herbicides [97]. In unicellular green algae, cytochrome P450 monooxygenases enable pre-herbicides’ N-demethylation (e.g., metflurazon), influencing sensitivity to these compounds [98]. Even nitroaromatic pollutants such as TNT can be broken down via nitrate reductases secreted by microalgae [99]. Together, these interconnected genetic and enzymatic networks reinforce the capacity of microalgae and cyanobacteria to serve as potent bioremediation agents for a broad spectrum of pesticide contaminants. The metabolism of pesticides in microorganisms and microalgae is a complex, multifaceted process (Figure 2), typically comprising functionalization (oxidation, reduction, hydrolysis), conjugation (e.g., glutathione, glucose, or organic acids), and compartmentalization (vacuolar or extracellular deposition) [100]. Key enzymes in these pathways include cytochrome P450-dependent monooxygenases, glutathione S-transferases, and glucosyltransferases [101], and microbial pesticide degradation may proceed via co-metabolism, conjugate formation, or accumulation.

The biodegradation of pesticides by microalgae is increasingly important, given their efficiency in detoxifying harmful organic compounds in various environments. Numerous studies indicate that microalgae perform well in pesticide biodegradation within aquatic systems. For example, using live cells, *Chlorella vulgaris* can eliminate 87–96.5% of pesticides in long-term experiments and 86–89% in short-term tests [59]. A microalgal consortium showed effective degradation of hydrophobic pesticides, with overall degradation—combining biodegradation and photodegradation—achieving removal rates of 55% for oxadiazon, 35% for chlorpyrifos, and 14% for cypermethrin [6]. Similarly, *Chlorella vulgaris* treatments reduced the total concentrations of several commonly used industrial pesticides—including carbofuran, carfentrazone-ethyl, fludioxonil, phenmedipham, propamocarb, and terbuthylazine, among others—from 63.5 µg/L to 29.7 µg/L over four days, compared to 37.0 µg/L in the control samples [102]. The principal mechanisms of pesticide removal are biosorption and biodegradation [64], although abiotic factors such as sunlight and aeration also help reduce pesticide content [102]. These findings underscore the potential of microalgae-based systems for purifying pesticide-contaminated waters.

These findings underscore the remarkable efficiency of bioremediation as an economically and ecologically viable strategy for detoxifying pesticide-contaminated sites. A thorough understanding of pesticide metabolism in microalgae is vital, not only for the design of safer chemical compounds but also for the development of effective ecological restoration methods. Deploying microalgae for the biodegradation of organic pollutants presents an innovative biotechnological solution to revitalize polluted or degraded areas, as well as to combat desertification in arid regions. Their aptitude for harnessing solar energy and degrading multiple pollutants confers notable advantages in remediating natural ecosystems.

Further research should optimize strain selection, reactor design, and operational parameters—such as nutrient ratios and cultivation conditions—to maximize pesticide removal. Metabolic engineering and pathway analyses can bolster specific degradation routes, particularly for stubborn pollutants. Integrating microalgal systems into conventional wastewater treatment or adopting modular photobioreactors could facilitate broader agricultural runoff and industrial effluents implementation. Optimizing these remediation approaches by selecting robust strains and refining application strategies will be critical for advancing eco-friendly pest management practices that detoxify soils, enhance fertility, and protect both human health and the environment.

## 4. Effect of Microalgae and Cyanobacteria on Soil Fertility

Microalgae significantly enhance soil fertility by improving soil structure, boosting nutrient availability, and stimulating beneficial microbial activity [103]. Multiple studies confirm that microalgae and their extracts elevate soil biological activity, biochemical indices, and plant growth [57]. As effective biofertilizers, they promote nutrient cycling, produce bioactive substances such as phytohormones, and support beneficial soil microorganisms [104]. Introducing cyanobacteria and microalgae provides advantages such as higher seed germination rates, improved grain yields, and better crop nutritional value [105]. Cyanobacteria can produce phytohormones—plant growth regulators such as auxins, cytokinins, and gibberellins—that promote plant growth and development. In addition, they contribute to nutrient mineralization, biocontrol, land reclamation, and strengthening plant defense mechanisms (Table 2). Specialized nitrogen-fixing cells supply nitrogen to soil microbes, macrofauna, and plants [106], significantly increasing soil nitrogen content [107] and allowing a 25–40% reduction in chemical nitrogen fertilizers [108]. For instance, nitrogen-fixing filamentous cyanobacteria can cut rice fertilizer use by 50% without affecting yield or grain quality [109], benefits also noted in vegetables, cotton, and cereals [110]. Species like *Nostoc entophytum* and *Oscillatoria augustissima* reduce fertilizer costs and increase seed nutrient value in peas [110], while an *Anabaena* sp. biofilm in wheat elevates soil nitrogen levels by 57.42% and 40% [111]. Additional studies highlight their positive impacts on water retention, organic carbon content, total nitrogen, microbial activity [112], and nutrient dynamics in soil [113,114]. Cyanobacteria and green algae are emerging as valuable bioinoculants, enhancing soil structure, nutrient cycling, and microbial activity, leading to higher yields [115] while adjusting soil pH and serving as biocontrol agents [116]. They produce phytohormones, exopolymers, and play a role in carbon sequestration and soil restoration [115,116,117,118].

Microalgae also improve soil aggregate stability and reduce erosion [119]. In sandy or polluted areas, algae initiate pedogenesis and form organic-rich crusts containing aliphatic/aromatic compounds, carbohydrates, and nitrogenous substances, plus higher mineral content [121]. *Chlamydomonas*, *Chlorella*, *Nostoc*, and *Oscillatoria* can enhance water resistance of soil aggregates, with *Chlamydomonas* inoculations increasing aggregate stability by 11–77% through polysaccharide production [119,120]. Soil water retention improves by 2–5% [120]. Under salinization and desertification, salt-tolerant microalgae decrease soil salinity, enhance soil properties, and boost yields in saline-alkaline areas [129] while removing pollutants from saline wastewater and creating valuable byproducts [130]. Biofertilizers derived from *Chlorella pyrenoidosa* increase yields of halophytes such as quinoa and microalgal eco-farms integrate soil amelioration, CO_2_ emission reduction, and agricultural production [131]. Nitrogen-fixing cyanobacteria and microalgae combat desertification by forming cryptogamic crusts that protect soil structure, contribute nitrogen, and boost biomass [135,136]. Combining cyanobacteria with soil-fixing chemicals and superabsorbent polymers accelerates crust formation and soil stability [137]. Inoculating local nitrogen-fixing strains like *Nostoc commune*, *Scytonema hyalinum*, and *Tolypothrix distortiona* raises organic carbon and total nitrogen in semi-arid soils [122]. Meanwhile, cyanobacteria rapidly form bio-crusts that reinforce soil resilience, with nitrogen-fixing strains especially effective at boosting organic carbon and nitrogen contents in sandy or clay soils [123].

Microalgae and cyanobacteria also maintain soil fertility and health by promoting plant growth, facilitating symbioses with other microbes, and detoxifying pollutants [117]. They exchange nutrients and signals with soil bacteria—providing nitrogen, vitamins, and hormones—and produce vitamin signals that affect bacterial quorum sensing [124]. Inoculating soil with microalgal suspensions stimulates microbial activity and forms stable communities [125], while cyanobacterial inoculants reshape rhizosphere microbiota involved in nutrient dissolution and mineralization [126]. Exopolysaccharides from cyanobacteria supply organic carbon, promote biofilm formation, reinforce mechanical stability, and facilitate nutrient flow [127,138]. These matrices incorporate inorganic (silica, carbonate) and organic (nucleic acids, proteins, polysaccharides) substances, providing essential carbon [128]. They also allow deeper light penetration for oxygen and carbon supply during nitrogen fixation and photosynthesis in green algae and cyanobacteria [139]. Both live microalgae and their extracts enhance soil biological activity, increasing soil fertility indices [57], while wet microalgal additives reduce nitrogen losses [138]. Furthermore, interactions with other microorganisms (e.g., *Azospirillum brasilense*) can boost ADP-glucose pyrophosphorylase activity in *Chlorella vulgaris*, raising starch content and carbon uptake [133,134]. Altogether, these findings emphasize the essential roles of microalgae and cyanobacteria in shaping soil health, nutrient flow, and valuable bioactive substances.

Overall, microalgae and cyanobacteria emerge as powerful bioinoculants that can strengthen soil fertility, mitigate salinity and desertification, and enhance crop yields—all while reducing reliance on chemical inputs. Their swift formation of protective bio-crusts, versatile metabolic processes for nutrient enrichment, and capacity to support beneficial microbial communities are significantly sustainable agriculture. By leveraging these microorganisms—through thoughtful strain selection, appropriate cultivation methods, and synergy with existing agroecological practices—farmers and land managers can significantly boost soil health and resilience, ultimately fostering higher productivity and a reduced environmental footprint. Continued research and innovation will be key to maximizing the potential of these remarkable soil allies for long-term agricultural and ecological sustainability.

## 5. Integration of Microalgae and Cyanobacteria into Sustainable Agriculture: Biomass Production and Bioremediation

Integrating microalgae and cyanobacteria into modern agricultural systems confers various benefits for sustainable agriculture, encompassing soil and wastewater treatment innovations and biomass production. These systems exploit the capacity of microalgae for nutrient sequestration [139], making them promising for environmental management. Cultivating microalgae and cyanobacteria does not require fertile land, large volumes of freshwater, or herbicides and pesticides, thereby avoiding resource competition [140]. Moreover, they can be grown using domestic wastewater [141]—a critical advantage in the face of global freshwater shortages caused by population growth, rising living standards, and increasing agricultural and industrial water demands [142]. By 2025, 48 countries could experience chronic water scarcity, affecting roughly 35% of the global population [142]. This challenge is compounded by wastewater pollution, necessitating effective treatment methods to protect public health and the environment [143]. Various solutions have been proposed, including desalination of brackish water and seawater, plus wastewater reclamation [144], while resource-saving and cost-effective treatments remain crucial [145]. Against this backdrop, cultivating microalgae and cyanobacteria in wastewater has garnered attention as a sustainable route for biomass production and wastewater remediation [146]. Such dual-purpose cultivation offers an economically viable strategy for large-scale biomass yields and efficient pollutant removal [147]. Phytoremediation using these photosynthetic microorganisms has thus emerged as an environmentally sound technology for eliminating wastewater contaminants [148]. Notably, studies suggest that certain microalgal strains grow as effectively in domestic wastewater as in synthetic media [149], reducing cultivation costs and facilitating simultaneous wastewater treatment and biomass harvest (Table 3).

The resulting biomass can be channeled into biofertilizer production—contributing up to 30 kg of nitrogen per hectare—and offers an eco-friendlier alternative to chemical fertilizers. Additionally, microalgal biomass can serve as a bioremediator for soil pesticide degradation, marking a promising bioremediation avenue [164]. Large-scale cultivation of microalgae and cyanobacteria frequently relies on open ponds or closed photobioreactors [165]. Open ponds, particularly channel ponds, remain a cost-effective and straightforward option despite being prone to environmental fluctuations and yielding lower productivity [166]. Photobioreactors, by contrast, are closed systems that offer superior control over culture conditions and typically achieve higher biomass productivity [167]. Various photobioreactor designs—tubular, airlift columns, flat panels, or mixed tanks—seek to optimize light usage, gas exchange, and mixing to stimulate microalgal growth [168,169]. Yet construction and maintenance expenses often limit commercial viability [170]. Recent work has focused on cutting costs via cheaper materials such as autoclaved polycarbonate [170]. Cultivation parameters (e.g., aeration rate, light intensity, inoculum density) also require optimization [170]. Some researchers propose integrating open and closed systems to balance production yields with operational expenses [171]. Regardless of the chosen approach, critical considerations include lighting, mixing, water and CO_2_ input, O_2_ removal, nutrient supply, temperature, and pH [171,172].

A crucial stage in microalgae and cyanobacteria cultivation is harvesting, which accounts for 20–30% of total production costs [173]. Standard methods—filtration, centrifugation, flotation, and flocculation—vary in efficiency and energy demand [174]. While centrifugation and membrane technologies can surpass 90% efficiency, flotation and flocculation typically reach 75–80% [175]. Energy usage ranges from 0.07 to 11.1 kWh/m^3^ of suspension [175], influenced by cell properties, culture density, and product specifications [173]. Despite extensive research, no universal collection method exists, given the diverse applications of microalgae and cyanobacteria (biofuels, food, high-value products, soil/water restoration) [164]. Thus, the chosen technique should align with the intended end-use and the required purity and yield of the final product. Following collection and preparation, microalgae and cyanobacteria can be employed for pesticide biodegradation in soil, as evidenced by field experiments demonstrating water and soil cleanup. In one investigation, photobioreactors achieved total removal for 10 of 51 tested pesticides [176]. Microalgae remove pesticides through diverse metabolic routes, and these efficiencies can be boosted via various interventions [7]. A *Chlorella vulgaris–Scenedesmus quadricuda–Spirulina platensis* consortium removed up to 99% of malathion while bioaccumulating heavy metals (nickel 95%, lead 89%, cadmium 88%) in wastewater [88]. Although removal rates may vary similarly to conventional treatment plants, microalgae-based systems can yield value-added products during remediation [7].

In addition to water decontamination, microalgae and cyanobacteria also show promise in soil bioremediation efforts—especially for organophosphorus pesticides—while aiding soil restoration in arid environments. Cyanobacteria such as *Nostoc commune* tolerate and degrade these compounds (e.g., malathion) by using them as a phosphorus source [75,177]. *N. commune* can be cost-effectively cultured in large bioreactors using fertilizer-based media [178], then introduced into degraded soils to raise organic carbon and nitrogen levels and improve soil functions [122]. By forming biofilms, these organisms boost soil fertility and combat erosion in arid ecosystems. Laboratory trials achieved 50% cyanobacterial coverage of various soil types within three months [122]. However, field-scale applications face obstacles, as changes in soil composition, climate, and microbial interactions can hinder direct inoculation success [179,180]. Researchers thus advocate habitat improvement measures (e.g., covering inoculated soils with plant nets), refined formulation methods, and smart delivery solutions to bolster efficacy [179,181]. A comprehensive strategy encompassing strain discovery, multifunctional traits, and fermentation approaches can strengthen the impact of microbial inoculants [181]. Ultimately, microalgae and cyanobacteria exhibit a remarkable ability to colonize various soil types and enhance soil fertility, establishing them as robust candidates for large-scale soil restoration and pesticide remediation [182]. These examples confirm that with proper collection and application methods, microalgae and cyanobacteria can effectively perform bioremediation, ensuring cleaner, safer environments.

Overall, integrating microalgae and cyanobacteria into agricultural systems presents a powerful approach to enhancing sustainability. By cultivating these organisms in open ponds, closed photobioreactors, or hybrid systems—often using wastewater streams as a resource—producers can address multiple challenges simultaneously. Microalgae and cyanobacteria help remove nutrients and toxicants from water, degrade pesticides in soil, and generate valuable biomass for biofertilizers, feed supplements, and other high-value products. Beyond environmental remediation, this approach can reduce reliance on chemical inputs, support carbon sequestration, and improve soil health—ultimately contributing to a circular bioeconomy that conserves water and energy resources.

Looking ahead, continued research and innovation can further boost the feasibility of large-scale microalgal and cyanobacterial cultivation. Cost-effective reactor designs, optimized harvesting techniques, and selective breeding or genetic engineering of robust strains will be key to raising biomass yields and expanding the range of contaminants targeted. Moreover, integrating these photosynthetic microorganisms into existing wastewater treatment infrastructures or constructing specialized photobioreactors in regions prone to desertification or salinization can unlock their full remediation potential. By embracing these multi-functional organisms, stakeholders can chart an optimistic path toward cleaner water, healthier soils, and more resilient agricultural ecosystems.

## 6. Economic and Environmental Benefits, Challenges, and Prospects of Using Microalgae and Cyanobacteria

Modern agricultural practices have long relied on pesticides to boost crop yields and meet growing food demands. However, this reliance has precipitated critical environmental and health concerns, particularly concerning organophosphorus pesticides that threaten soil microbes, human health, and the food chain through their toxicity and persistence [183]. An estimated 3 billion kilograms of pesticides are applied annually, leading to ecological imbalances and adverse organ effects in humans, including potential cancer risks [1]. These compounds can remain in the environment for extended periods, causing bioaccumulation and disturbing ecosystem equilibrium [1]. Simultaneously, intensive farming practices have contributed to soil fertility depletion and heavy metal buildup [183]. More sustainable solutions have been proposed, such as organic and biodynamic farming [1] and bioremediation leveraging bacteria, microalgae, fungi, and plants [183].

Soil algalization—applying microalgae and cyanobacteria to improve soil conditions—has demonstrated considerable success. Field results indicate that algalization as a plant biostimulant has yielded favorable outcomes in various Indian states as well as in China, Egypt, Vietnam, and the Philippines [184]. Microalgae also serve as soil quality and pollution indicators [185]. Acid-resistant strains have shown the ability to increase soil carbon content and secrete exopolysaccharides and indole-3-acetic acid [154]. Recent research on bioalgalization—using *Spirulina* with biofertilizers and organic matter—demonstrates significant improvements in nitrogen, phosphorus, and potassium levels, leading to higher yields and increased protein content [151]. These findings suggest that algalization can bolster soil fertility while minimizing chemical fertilizer dependence. Moreover, microalgae have proven promising in bioremediating pesticides and contaminated habitats, removing pesticides through mechanisms like biodegradation, photodegradation, and sorption [7]. They can degrade organophosphates, pyrethroids, and other classes of pesticides [6], and their introduction into soil raises species diversity, organic matter, and cation exchange capacity, thereby potentially restoring soil quality after herbicide use [186].

Microbial pesticide degradation is an economical, eco-friendly remediation strategy that curtails environmental toxicity and revives natural cycles [45]. Microbes adapt to environments containing pesticides over time, heightening their degradation capabilities [45]. This process depends on pesticide properties, environmental factors, and microbial characteristics [187]. Within this framework, microalgae and cyanobacteria hold promise for reviving polluted ecosystems, improving water and soil quality, fostering biodiversity, and elevating ecosystem resilience. Their biodegradation of pesticides diminishes toxicity levels, helping restore ecological balances and preserve vital ecosystem services.

Despite these advantages, employing microalgae and cyanobacteria for pesticide biodegradation faces notable limitations. Chief among them are the need to optimize growth conditions, the challenges of scaling up to industrial levels, and substantial upfront costs. Large-scale production complicates contamination control and harvesting strategies [172]. Contamination risks rise with increased culture volumes, and harvesting methods—from auto flocculation to centrifugation and filtration—can account for significant energy use and financial investment [188]. Resolving these scale-up hurdles is essential to harnessing the full potential of microalgae for biofuels, bioproducts, and soil and water bioremediation [188].

Developing new microalgal strains with enhanced pesticide degradation abilities and refining growth conditions remain critical objectives. Integrating biodegradation techniques into existing waste treatment infrastructures is another promising direction [58]. Future systems may merge various methods to increase efficiency and broaden the scope of bioremediation applications [189]. Market trends point to rapid growth in microalgae-based products, projected to reach USD 1.92 billion in 2024 and USD 2.45 billion by 2029, with a compound annual growth rate of 4.99% [190]. This expansion—paired with the rising popularity of organic farming, likely to exceed 75 million hectares—signals growing awareness, ecological benefits, and potentially supportive policies that could stimulate commercial-scale production of biofertilizers, biostimulants, and pesticide-degrading agents.

In addition to academic research, several companies worldwide are currently advancing the real-world application of microalgae in environmental management. FCC Aqualia [191] operates Europe’s largest wastewater-to-biofertilizer facility, showcasing how algal-based systems can contribute to mitigating pesticide runoff and nutrient pollution. AlgaEnergy [192] has developed microalgae-derived agricultural biostimulants that reduce reliance on chemical fertilizers and pesticides, while Microphyt [193] demonstrates large-scale microalgal cultivation platforms with potential applications in agriculture and soil restoration. In the United States, Heliae’s PhycoTerra [194] products improve soil health through microalgal amendments, and Cyanotech [195] exemplifies the feasibility of industrial-scale algal production. Together, these industrial efforts illustrate the capacity of microalgae to bridge laboratory findings with practical solutions, while also highlighting remaining challenges such as scalability, cost-effectiveness, and integration into existing agricultural frameworks.

Nevertheless, the contribution of microalgae and cyanobacteria in conventional agriculture remains limited, primarily due to industry hesitance and the complexities of large-scale integration. Yet this approach holds significant promise for more sustainable farming systems, offering reductions in agrochemical expenses and improved financial stability in agriculture. While laboratory studies have validated the potential of these microorganisms for pesticide cleanup, further pilot studies are necessary to translate these findings into practical, commercially viable solutions [196]. Overcoming technological and economic challenges in culturing, harvesting, and applying microalgae will be vital for realizing this vision. Successful deployment of microalgae and cyanobacteria for bioremediation could dramatically enhance environmental conditions while curbing reliance on chemical inputs, ultimately strengthening both ecosystem health and agricultural profitability.

## 7. Conclusions

Microalgae and cyanobacteria hold immense potential for tackling pressing environmental challenges, particularly in mitigating soil and wastewater pollution. Leveraging these microorganisms for algalization processes and pesticide biodegradation promotes more efficient wastewater treatment and reinforces soil fertility by enhancing soil structure, mineralizing nutrients, and utilizing their catabolic genes for pollutant breakdown. Integrating microalgae and cyanobacteria into existing wastewater management frameworks and applying their biomass to soil effectively diminishes pollutant loads, restores ecosystem function, and reduces overall treatment expenses. Additionally, repurposing wastewater as a nutrient-rich growth medium transforms a liability into a valuable resource, lowering costs and driving agricultural sustainability.

Broader implementation requires robust research, committed funding, and international collaboration despite these clear advantages. Scaling up cultivation systems, optimizing strain selection, and developing cost-effective harvesting methods remain critical hurdles. Addressing these needs with targeted support will expedite the adoption of microalgae- and cyanobacteria-based technologies, delivering long-term environmental gains while boosting soil health and productivity. This dual role in waste management and soil restoration, supported by their metabolic versatility, underscores why microalgae and cyanobacteria warrant heightened global attention and investment as we strive for safer, more resilient agroecosystems.

## Figures and Tables

**Table 1:** Mechanisms and pathways of pesticide and pollutant degradation by microalgae and cyanobacteria.

Mechanism/Pathway	Organism(s) Studied	Target Pollutant(s)	Key Enzymes/Genes/ Processes	References
Degradation of organophosphate pesticide (hydrolysis and transformation)	Microalgae and cyanobacteria	Methyl parathion	Hydrolysis and subsequent metabolic transformation (specific enzymes not identified)	[8]
Aromatic hydrocarbon degradation	Indigenous soil microbial consortia (diesel-contaminated soil)	Diesel hydrocarbons	Catabolic genes xylE, ndoB	[51]
Reductive dechlorination via nitrate-reduction system	Filamentous cyanobacteria	Lindane	*nir* operon (nitrate reduction), light-dependent, ammonium inhibition	[52]
Mixotrophic carbon assimilation and pollutant degradation	Mixotrophic cyanobacteria and microalgae	Aromatic hydrocarbons, pesticides	Mixotrophic growth, pollutant adsorption; potential enzymatic transformation	[11,53]
Phytoremediation of wastewater pollutants	Various microalgae and cyanobacteria	Organic pollutants in wastewater	Adsorption, enzymatic transformation	[54,55]
Degradation of phenolic and heterocyclic aromatic compounds	*Lyngbya lagerlerimi*, *Nostoc linckia*, *Oscillatoria rubescens*, *Elkatothrix viridis*, *Volvox aureus*	Phenols, PAHs	Phenol oxidation to catechol; further degradation observed	[56]
Plastic degradation potential	Microalgae, cyanobacteria	Plastics	Oxidation, enzymatic breakdown	[57]

**Table 2:** Effects of Microalgae and Cyanobacteria on Soil Fertility.

Effect Category	Mechanism/Microorganisms	Observed Outcomes/ Benefits	References
Soil structure and stability	*Chlamydomonas*, *Chlorella*, *Nostoc*, *Oscillatoria*; polysaccharides	↑ Aggregate stability (11–77%), ↓ erosion, improved pedogenesis	[119,120,121]
Nutrient availability and mineralization	Nitrogen-fixing cyanobacteria (*Anabaena* sp., *Nostoc*, *Scytonema*, *Tolypothrix*), microalgae	↑ Soil N (up to 57%), ↑ total N and organic C, reduce chemical N fertilizers by 25–50%	[106,107,109,110,111,122,123]
Plant growth promotion	Cyanobacteria, microalgae; phytohormones (auxins, cytokinins, gibberellins)	↑ Seed germination, ↑ yield, ↑ grain and crop nutritional value	[104,105,115]
Soil microbial activity and symbiosis	Microalgal suspensions, cyanobacterial biofilms; exopolysaccharides	↑ Soil microbial activity, stable microbial communities, nutrient cycling, enhanced rhizosphere microbiota	[117,124,125,126,127,128]
Water retention and salinity mitigation	*Chlamydomonas*, salt-tolerant microalgae	↑ Soil water retention (2–5%), ↓ salinity, ↑ yields in saline-alkaline soils	[120,129,130,131]
Bioactive compound production	Microalgae and cyanobacteria	Production of phytohormones, exopolymers, bioactive metabolites, carbon sequestration	[57,116,117,118,132,133,134]

**Table 3:** Biomass Production by Microalgae Cultivated in Different Types of Wastewaters.

Wastewater	Microalgae	Biomass Accumulation (DCW g/L/day)	References
Municipal wastewater	*Chlorella* sp.	0.92	[124]
Industrial wastewater	*Chlamydomonas* sp. *TAI-2*	0.134	[13]
Municipal wastewater	*Chlorella* sp. *ADE5*	3.01	[150]
Municipal wastewater	*Chlorella vulgaris*	0.039–0.195	[151]
Piggery wastewater	*Chlorella zofingiensis*	1.314	[152]
Urban wastewater	*Desmodesmus communis*	0.138–0.227	[153]
Municipal wastewater	*Chlorella sorokiniana*	0.22	[154]
Municipal wastewater	*Dunaliella salina*	0.1695	[155]
Aquaculture wastewater	*Chlorella minutissima*	4.77	[156]
Municipal wastewater	*Tetraselmis* sp. *NKG400013 Parachlorella kessleri NKG021201* *Chloroidium saccharophilum*	1.03	[157]
Swine wastewater	*Chlorella sorokiniana AK-1*	5.45	[158]
Municipal wastewater	*Scenedesmus obliquus*	0.58	[159]
Dairy wastewater	*Chlorella vulgaris* (C.), *Scenedesmus* (S.), *Tribonema* (T.), *Lyngbya* (L.) (consortium S:T)	0.084	[160]
Urban wastewater	*Chlorella* sp.	0.30	[161]
Dairy wastewater	*Spirulina platensis Micractinium*, *Chlorella*	0.251	[162]
Dairy wastewater	*Limnospira platensis*	0.330	[163]

## Data Availability

No new data were created or analyzed in this study. Data sharing is not applicable to this article.
